# Variation in Medicare Part D and Part B drug spending among older adults with Alzheimer’s disease and related dementias

**DOI:** 10.1093/geroni/igag085

**Published:** 2026-08-03

**Authors:** Seyeon Jang, Jie Chen

**Affiliations:** Department of Health Policy and Management, University of Maryland School of Public Health, College Park, Maryland, United States; Center for Seniors Uniting Nationwide to Support Health, INtegrated care, and Economics (SUNSHINE), University of Maryland, College Park, Maryland, United States; Department of Health Policy and Management, University of Maryland School of Public Health, College Park, Maryland, United States; Center for Seniors Uniting Nationwide to Support Health, INtegrated care, and Economics (SUNSHINE), University of Maryland, College Park, Maryland, United States

**Keywords:** Medicare drug spending, Drug costs, Alzheimer’s disease and related dementias, Drug cost burden

## Abstract

**Background and Objectives:**

This study examined Medicare Part D and Part B drug spending and beneficiary out-of-pocket (OOP) costs among community-dwelling Medicare fee-for-service (FFS) beneficiaries with Alzheimer’s disease and related dementias (ADRD), assessing variation by dual eligibility, state, and Accountable Care Organization (ACO) enrollment.

**Research Design and Methods:**

We conducted a cross-sectional analysis using the 2022 Medicare Beneficiary Summary File linked to the Cost and Use file. The sample included 1 372 696 community-dwelling Medicare FFS beneficiaries with ADRD, including 575 308 beneficiaries with five or more chronic conditions. Outcomes included annual Medicare payments and beneficiary OOP costs for Parts D and B drugs. Analyses were stratified by dual eligibility duration, state, and ACO enrollment, with adjusted estimates from generalized linear models.

**Results:**

Medicare Part D drug spending was substantial, increasing with dual-eligibility duration: $8732-$9107 among beneficiaries with full-year dual eligibility and exceeding $11 500 among those with 5+ chronic conditions. Beneficiary OOP costs were highest among Medicare-only beneficiaries and markedly lower among dually eligible individuals. Medicare Part B drug spending was highest among Medicare-only beneficiaries and varied widely by state, ranging from $433 to $1827 among Medicare-only beneficiaries and from $179 to $1446 among dually eligible beneficiaries. After adjustment, differences in Parts D and B drug spending by ACO enrollment were modest.

**Discussion and Implications:**

Parts D and B drug costs among Medicare beneficiaries with ADRD vary by dual eligibility and geography. Limited associations with ACO enrollment underscore the need for better medication management integration in value-based care for high-need older adults with ADRD.

Innovation and Translational Significance: This study examines medication affordability and access among older adults with Alzheimer’s disease and related dementias (ADRD) by analyzing Medicare Part D and Part B drug spending and beneficiary out-of-pocket costs. Using linked Medicare claims, we assess variation by dual eligibility, state context, and Accountable Care Organization enrollment. The findings reveal substantial geographic and coverage-based differences—particularly for Part B drugs—and modest associations with ACO participation, highlighting structural gaps in medication management and informing policy and practice efforts to improve equitable access and care coordination in later life.

## Background and objectives

Individuals with Alzheimer’s disease and related dementias (ADRD) have substantial prescription drug needs, and many experience polypharmacy due to a high prevalence of coexisting chronic conditions.^1^^,^^2^ Compared with those without ADRD, beneficiaries with ADRD incur markedly higher prescription drug costs, spending nearly $3700 more among individuals in the top decile of drug spending.^3^ For general internists and other clinicians who provide longitudinal care for older adults with cognitive impairment, medication burden is a central driver of adverse drug events, treatment nonadherence, caregiver strain, care fragmentation, and potentially avoidable acute care use.

Recent advances in disease-modifying therapies for ADRD, including anti-amyloid monoclonal antibodies, have further heightened attention to medication management and drug-related spending in this population. These therapies require substantial outpatient clinical infrastructure, including infusion services, imaging, and ongoing monitoring for adverse events, and are typically covered under Medicare Part B rather than Part D.^4^ As a result, medication costs and care coordination responsibilities increasingly shift to office-based and specialty care settings, compounding existing challenges for individuals with ADRD who often have multiple chronic conditions and limited access to specialized services.

Although most prior studies of medication use among people with ADRD have focused on Medicare Part D, understanding Part B medication patterns is increasingly important because Part B drug spending reflects access to outpatient clinical care, specialty services, and the infrastructure required to deliver monitored, provider-administered therapies. Even in the absence of widespread uptake of dementia-modifying therapies in 2022,^4^ Part B drug use among people with ADRD serves as a meaningful indicator of access to outpatient care and system readiness for managing complex medication regimens, including infusion-based ADRD therapies. Examples of Part D and Part B medications used by beneficiaries with ADRD are provided in Supplementary Table 1.

Accountable Care Organizations (ACOs) are a cornerstone of value-based payment reform and have demonstrated success in reducing preventable hospitalizations, readmissions, and other forms of avoidable utilization.^5–7^ However, evidence on the role of ACOs in prescription drug use remains limited. Although medication management is not typically a primary focus of ACOs and ACOs are generally not financially accountable for Medicare Part D spending,^8^ they may influence medication use indirectly by promoting care coordination, facilitating access to appropriate therapies, and encouraging cost-effective prescribing practices.^8–10^ In contrast to Part D, ACOs are accountable for Medicare Part B spending and broader outpatient care coordination. This raises the possibility that ACO participation may influence access to provider-administered medications for people with ADRD. Whether ACO enrollment is associated with differences in Part B drug spending or beneficiary out-of-pocket (OOP) costs among individuals with ADRD remains largely unexplored.

Approximately 24% of community-dwelling adults with dementia are dually enrolled in Medicare and Medicaid.^11^ Although dual eligibility reduces beneficiary OOP drug costs through Medicaid cost-sharing assistance and low-income subsidies, even modest OOP expenses may remain burdensome for financially vulnerable beneficiaries.^12^^,^^13^ Dual-eligible beneficiaries also tend to have poorer health and greater healthcare needs than Medicare-only beneficiaries,^11^^,^^12^ potentially shifting medication-related costs from patients to Medicare and Medicaid programs. Coverage policies and cost-sharing protections also vary across states, contributing to potential geographic variation in access and spending.^12–14^ Moreover, the duration of dual eligibility may further influence medication-related spending and financial burden, as beneficiaries with partial-year dual eligibility may experience interruptions in Medicaid coverage, subsidy eligibility, and cost-sharing protections.^12^ These factors add complexity to the interaction between Medicare fee-for-service (FFS) coverage, dual eligibility, and ACO participation, particularly as high-cost, provider-administered medications increase. Yet little is known about how Part B medication spending varies by dual status, dual eligibility duration, and ACO enrollment among people with ADRD, or how state-level variation may shape financial burden and access to outpatient therapies.

In this study, we examine Medicare Part D and Part B drug spending and beneficiary OOP costs among community-dwelling Medicare FFS beneficiaries with ADRD, with a focus on those with coexisting chronic conditions such as hypertension, diabetes, and depression. We conceptualize Part D spending as reflecting polypharmacy burden and Part B spending as reflecting access to office-based and provider-administered therapies. Although disease-modifying dementia therapies were not widely reflected in 2022 claims, Part B drug spending provides a baseline measure of access and system readiness for emerging infusion-based ADRD therapies. We assess whether ACO participation is associated with differences in Part D and Part B drug costs, recognizing that improved access to necessary therapies may increase spending, while more effective care coordination and deprescribing may reduce costs.^8–10^^,^^15^^,^^16^ We further stratify the results by dual-eligibility status and examine state-level variation.

## Research design and methods

### Data

We use the 2022 Medicare Beneficiary Summary File (MBSF), linked with the MBSF Cost and Use file and the Medicare Shared Savings Program (MSSP) ACO beneficiary-level file. Medicare beneficiaries with ADRD and their chronic conditions are identified using the CMS’ Chronic Conditions Data Warehouse (CCW) algorithms.^17^ The study sample includes 1 372 696 community-dwelling Medicare beneficiaries with ADRD who were continuously enrolled in FFS for 12 months. The subgroup sample focuses on 575 308 beneficiaries with five or more chronic conditions other than ADRD. Beneficiaries are classified as community dwelling if they have fewer than 21 days in Medicare-covered skilled nursing facility stays during the year, consistent with Medicare’s full coverage threshold.^18^

### Outcome variables

The primary outcomes are annual Medicare payments and beneficiary OOP costs for Part D and Part B drugs (eg, physician-administered drugs, outpatient infusion drugs billed under Part B). Part D Medicare payments include low-income cost-sharing subsidies but exclude rebates and discounts.^19^ All Medicare payments are inflation-adjusted to 2023 dollars using the Personal Consumption Expenditures Health index, and beneficiary OOP costs are adjusted using the Consumer Price Index for medical care.^20^

### Key independent variables

The primary independent variable is full-year enrollment in an MSSP ACO. Beneficiaries who are never enrolled in an ACO or who have partial-year enrollment are classified as non-ACO.

### Covariates

Covariates include age, sex, race and ethnicity, full-year Part D enrollment, and the number of chronic conditions. Chronic conditions include diabetes, cardiovascular disease, depression, hypertension, cancer, asthma, arthritis, osteoporosis, hyperlipidemia, and chronic obstructive pulmonary disease. The cutoff of five or more chronic conditions is selected to reflect greater care complexity and increased limitations in instrumental activities of daily living.^21^ State fixed effects are included in all analyses to account for state-level variation.

### Analysis

We summarize Medicare payments and beneficiary OOP payments for FFS beneficiaries with ADRD and the subgroup with five or more chronic conditions.^21^ The analysis focuses exclusively on Medicare payments and excludes Medicaid or other payer data. We then examine Part D and Part B drug payments by ACO enrollment status and dual eligibility duration, categorized as 0, 1-11, and 12 months. Finally, we rank all U.S. states and the District of Columbia by average Medicare payment and highlight the five highest- and lowest-spending states.

We estimate adjusted Medicare payments using generalized linear models with a log link and gamma distribution to account for the skewed distribution of drug spending. All models adjust for age, sex, race and ethnicity, number of chronic conditions, and state fixed effects. We conduct sensitivity analyses using alternative definitions of ACO enrollment (full-year vs never enrolled) and alternative model specifications, and two-part models for OOP outcomes to account for zero-cost observations (See Supplementary Tables 2-5); results remain consistent across these analyses.

## Results

The study sample included 1 372 696 Medicare FFS beneficiaries with ADRD. Among them, 988 357 (72.0%) beneficiaries were not dually eligible during the year, 34 931 (2.5%) were dually eligible for 1-11 months, and 349 408 (25.5%) were dually eligible for the full 12 months. A total of 575 308 beneficiaries had five or more coexisting chronic conditions, including 388 051 (67.5%) with no months of dual eligibility, 14 378 (2.5%) with 1-11 months of dual eligibility, and 172 879 (30.0%) with 12 months of dual eligibility.

A summary of sample characteristics by ACO enrollment and dual eligibility durations is available in Supplementary Table 6. Compared with FFS-only beneficiaries, beneficiaries with longer dual-eligibility duration were more likely to be female, racial and ethnic minorities, and have a higher comorbidity burden.

**Figure 1:**
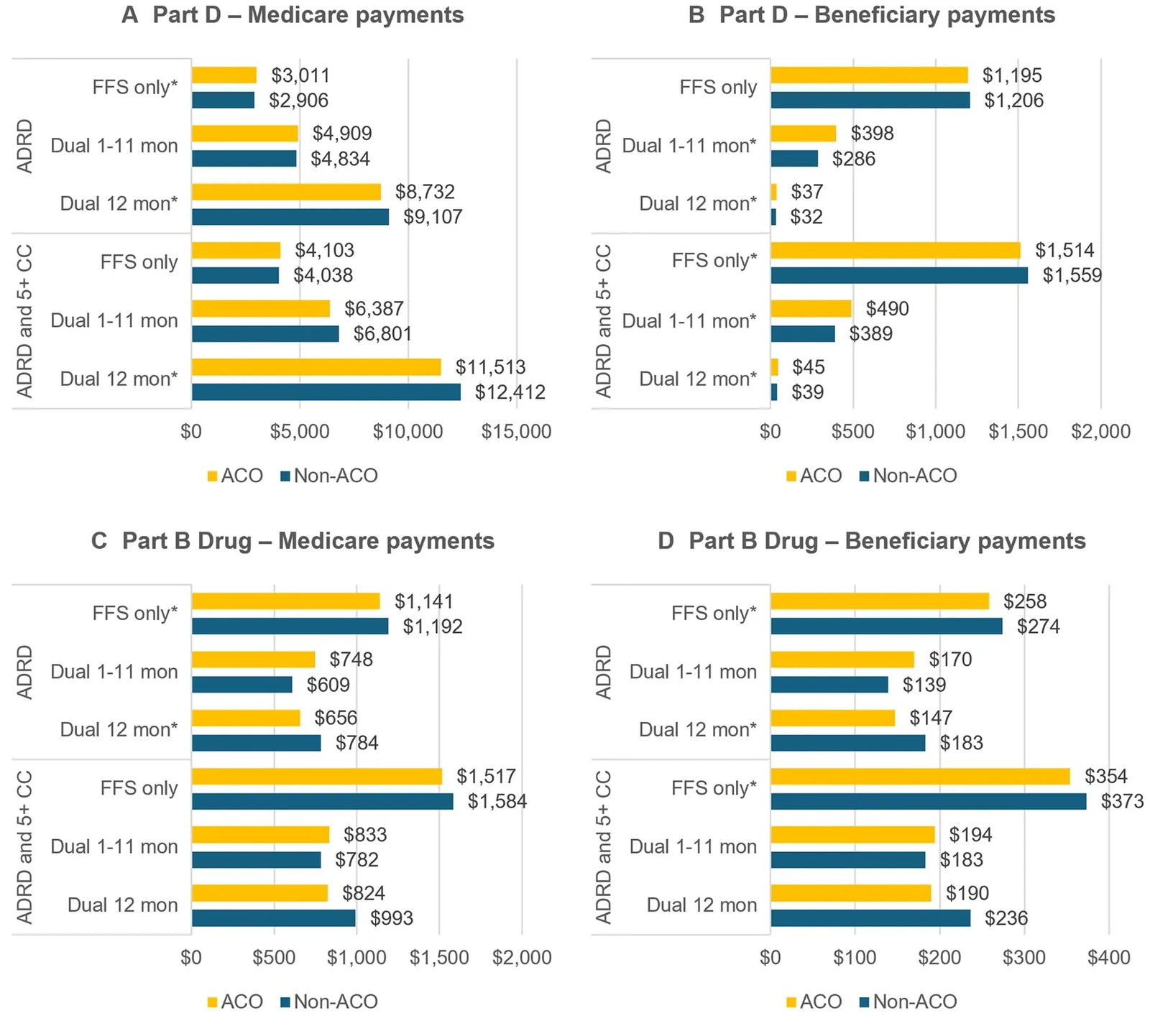
Unadjusted Part D and Part B Drug Payments among Community-dwelling FFS Beneficiaries with ADRD by Payer and Coverage Levels. ACO, accountable care organization; ADRD, Alzheimer’s disease and related dementias; CC, chronic conditions; Dual 1-11 months, dually eligible for 1 to 11 months; Dual 12 months, dually eligible for 12 months; FFS only, fee-for-service only. Bar graphs compare unadjusted Part D and Part B drug Medicare and beneficiary payments between ACO and non-ACO enrollees across different lengths of dual eligibility among ADRD beneficiaries and those with five or more chronic conditions. Medicare payments were adjusted for 2023 U.S. dollars using the Personal Consumption Expenditure Health Index, and beneficiary payments were adjusted using the Consumer Price Index for medical care. The non-ACO group includes beneficiaries who were never enrolled in an ACO and those with partial enrollment. *Indicates that payments were significantly different at *p *< .05.

Figure 1 (Panel A) shows that unadjusted Medicare Part D drug payments increased with dual eligibility duration.

Among FFS beneficiaries without dual eligibility, average Part D Medicare payments were similar between ACO and non-ACO enrollees ($3011 vs $2906). Among beneficiaries with 1-11 months of dual eligibility, Medicare payments increased to approximately $4800-$4900. For beneficiaries with full-year dual eligibility, payments exceeded $8700-$9100. Among beneficiaries with ADRD and five or more chronic conditions, Medicare Part D payments increased further, reaching $12 412 for non-ACO and $11 513 for ACO beneficiaries with 12 months of dual eligibility.

Panel B of Figure 1 summarizes unadjusted Part D drug beneficiary OOP payments. Average OOP payments among FFS-only beneficiaries were $1206 for non-ACO and $1195 for ACO enrollees. OOP costs declined substantially among beneficiaries with 1-11 months of dual eligibility. Among those with full-year dual eligibility, OOP payments dropped further to $32 and $37, respectively. Similar patterns were observed among beneficiaries with ADRD and five or more chronic conditions.

Panel C presents unadjusted Medicare Part B drug payments. Among FFS beneficiaries without dual eligibility, average Part B payments were $1192 for non-ACO and $1141 for ACO enrollees. Among beneficiaries with 12 months of dual eligibility, Part B payments were lower overall, averaging $656 for non-ACO and $784 for ACO beneficiaries. For individuals with ADRD and five or more chronic conditions, Part B drug payments were generally higher, with the highest payments observed among FFS-only beneficiaries ($1517 for non-ACO and $1584 for ACO).

Unadjusted Part B drug beneficiary OOP payments (Panel D) were approximately $260-$270 among FFS-only beneficiaries and lower among beneficiaries with 12 months of dual eligibility. Among individuals with ADRD and five or more chronic conditions, beneficiary Part B OOP payments were approximately $100 higher for FFS-only beneficiaries and about $50 higher for those with partial or full dual eligibility.

Table 1 presents Medicare Part D and Part B drug payments and beneficiary OOP payments by state. Among FFS-only beneficiaries, average Medicare Part D payments ranged from $2004 in South Dakota to $3561 in New York. Among beneficiaries with 1-11 months of dual eligibility, payments ranged from $2020 in New Mexico to $10 083 in the District of Columbia. For beneficiaries with full-year dual eligibility, the lowest spending was observed in Hawaii ($4843), while the highest spending states were New York ($12 206), California ($11 965), and New Jersey ($8827).

**Table 1:** The five lowest and highest spending states: Average Part D, Part B Drug spending per person among community-living FFS Medicare beneficiaries with ADRD by state.

		Part D total		Part B total		Part D OOP		Part B OOP	
**1-1. FFS only**									
** Lowest spending**	1	South Dakota	2004.25	Vermont	433.04	Wyoming	938.27	Vermont	86.39
	2	Wyoming	2047.39	Hawaii	520.03	Arkansas	963.39	Hawaii	100.44
	3	New Mexico	2147.65	Massachusetts	542.45	Montana	986.03	Massachusetts	107.77
	4	North Dakota	2148.64	New Hampshire	612.31	Utah	995.96	Rhode Island	130.49
	5	Minnesota	2215.51	Rhode Island	615.38	Maine	1009.73	New Hampshire	131.81
** Highest spending**	1	New York	3561.31	Nevada	1827.26	Alaska	2357.83	Nevada	448.38
	2	California	3300.92	Florida	1753.11	New York	1567.51	Florida	420.36
	3	New Jersey	3258.79	Mississippi	1566.55	Hawaii	1497.67	Mississippi	380.02
	4	South Carolina	3254.96	Texas	1531.43	Massachusetts	1396.04	Texas	367.92
	5	Vermont	3211.04	Arizona	1486.45	South Carolina	1346.20	Arizona	354.95
**1-2. Dually eligible for 1 to 11 months**									
** Lowest spending**	1	New Mexico	2020.29	Hawaii	199.83	Missouri	60.49	Hawaii	22.55
	2	Hawaii	2027.98	Utah	240.57	Montana	75.14	Alaska	36.77
	3	Oregon	2096.21	Alaska	252.24	Nevada	98.17	Utah	45.98
	4	Idaho	2469.58	New Hampshire	262.80	Colorado	210.06	Wyoming	50.71
	5	Utah	2538.32	South Dakota	269.21	Kansas	219.30	New Hampshire	50.87
** Highest spending**	1	DC	10 083.21	Nevada	1853.94	New Jersey	566.65	Nevada	462.40
	2	Missouri	8189.04	Mississippi	1795.96	Delaware	553.08	Mississippi	451.95
	3	Kansas	6476.81	Maryland	1667.77	Ohio	495.94	Maryland	403.30
	4	Montana	6187.96	Louisiana	1407.12	Rhode Island	494.34	Louisiana	347.39
	5	Maryland	5398.59	Georgia	1100.10	Iowa	488.45	Georgia	264.09
**1-3. Dually eligible for 12 months**									
** Lowest spending**	1	Hawaii	4842.55	Vermont	179.07	Colorado	12.51	Vermont	33.93
	2	New Mexico	5060.67	New Hampshire	220.40	Iowa	12.65	New Hampshire	45.01
	3	Rhode Island	5626.24	Montana	250.79	Minnesota	13.47	South Dakota	45.27
	4	Vermont	5777.50	South Dakota	263.92	Kansas	13.88	Montana	48.21
	5	Maine	5919.17	Hawaii	288.45	New Hampshire	14.08	Hawaii	53.91
** Highest spending**	1	New York	12 205.87	Nevada	1445.81	Vermont	163.86	Nevada	362.18
	2	California	11 964.65	Mississippi	1378.85	North Carolina	67.35	Mississippi	345.51
	3	New Jersey	8826.63	California	1172.90	Maine	62.89	California	283.35
	4	Kentucky	8648.86	Arizona	1166.10	Nevada	52.30	Arizona	281.56
	5	Oklahoma	8306.54	Missouri	1132.62	California	50.84	Missouri	277.41

Payments are the average Part D and Part B drug Medicare and beneficiary payments per person among FFS beneficiaries with ADRD, by state, across three groups: FFS-only, dually eligible for 1 to 11 months, and dually eligible for 12 months.

Abbreviations: ADRD, Alzheimer’s disease and related dementias; DC, District of Columbia; FFS, fee-for-service.

Substantial geographic variation was also observed in Medicare Part B drug payments. Among FFS-only beneficiaries, average payments ranged from $433 in Vermont to $1827 in Nevada and $1753 in Florida. Among beneficiaries with 12 months of dual eligibility, Part B payments ranged from $179 in Vermont to $1446 in Nevada.

Beneficiary OOP payments also varied widely across states. For Part D drugs among FFS-only beneficiaries, average OOP payments ranged from $938 in Wyoming to $2358 in Alaska. Among beneficiaries with full-year dual eligibility, Part D OOP payments were substantially lower, ranging from $13 in Colorado to $164 in Vermont. For Part B drugs, beneficiary OOP payments ranged from $86 in Vermont to $448 in Nevada among FFS-only beneficiaries and from $34 in Vermont to $362 in Nevada among beneficiaries with 12 months of dual eligibility.

**Figure 2:**
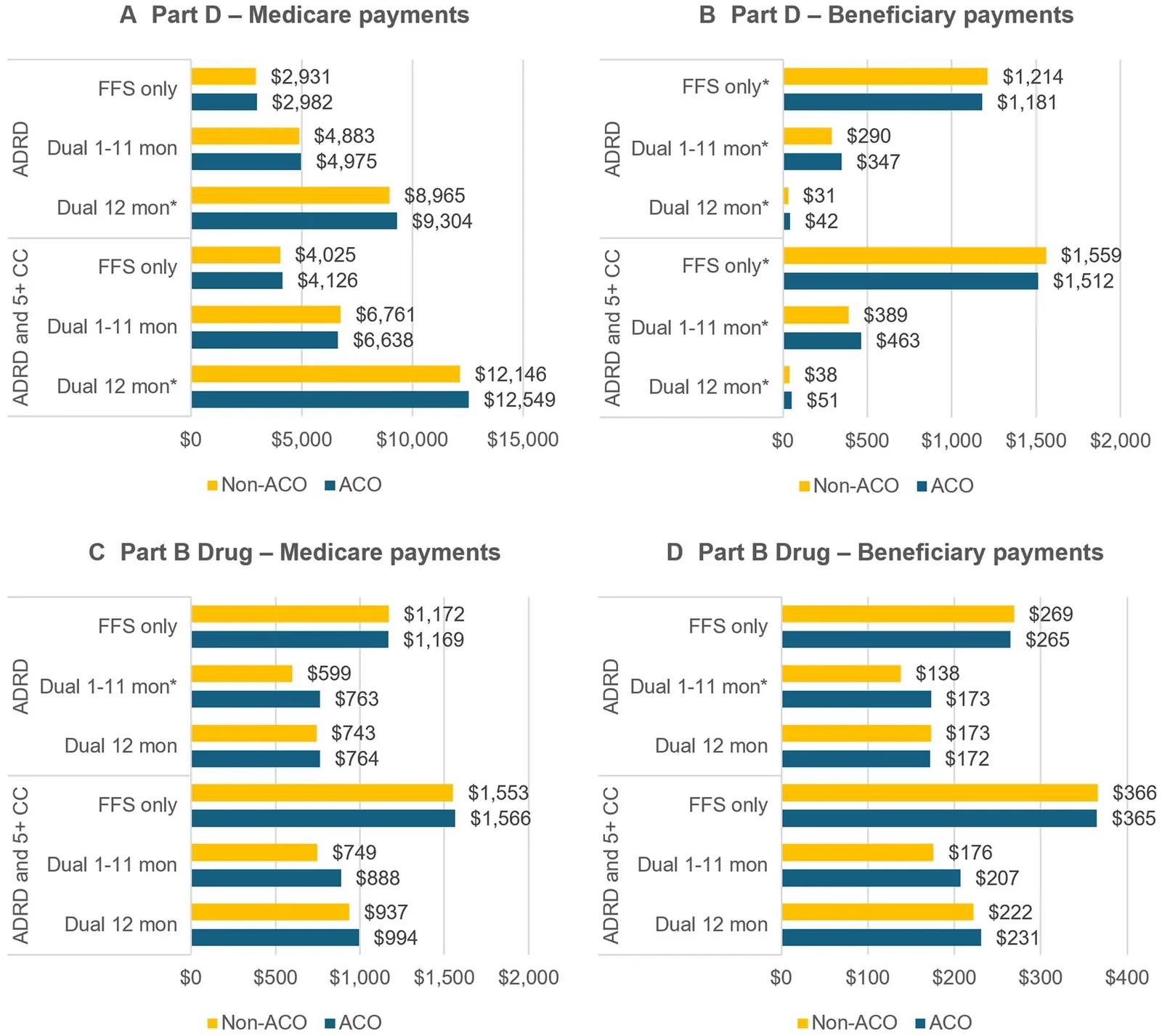
Adjusted Part D and Part B Drug Payments among Community-dwelling FFS Beneficiaries with ADRD by Payer and Coverage Levels. ACO, accountable care organization; ADRD, Alzheimer’s disease and related dementias; CC, chronic conditions; Dual 1-11 months, dually eligible for 1 to 11 months; Dual 12 months, dually eligible for 12 months; FFS only, fee-for-service only. Bar graphs compare adjusted Part D and Part B drug Medicare and beneficiary payments, controlling for age, sex, race/ethnicity, Part D coverage, and the number of comorbidities, between ACO and non-ACO enrollees across different lengths of dual eligibility among ADRD beneficiaries and those with five or more chronic conditions. The non-ACO group includes beneficiaries who were never enrolled in ACO and those with partial enrollment. *Indicates that payments were significantly different at *p *< .05. Full regression results underlying the predicted payment estimates are available in Supplementary Tables 7-14.

Figure 2 (Panel A) presents adjusted Medicare Part D payments by ACO enrollment status. Adjusted Part D Medicare payments were lowest among FFS-only beneficiaries ($2931 for non-ACO and $2982 for ACO), increased among beneficiaries with 1-11 months of dual eligibility, and were highest among beneficiaries with 12 months of dual eligibility ($8965 for non-ACO and $9304 for ACO). Similar patterns were observed among beneficiaries with ADRD and five or more chronic conditions. Adjusted Part B drug Medicare payments followed comparable trends (Panel C). Panel B and Panel D of Figure 2 present adjusted beneficiary OOP payments, which were highest among FFS-only beneficiaries and lowest among beneficiaries with full-year dual eligibility, with minimal differences by ACO enrollment status.

## Discussion and implications

This study provides a comprehensive assessment of medication-related spending among community-dwelling Medicare FFS beneficiaries with ADRD by examining Medicare Part D and Part B drug payments, stratified by dual eligibility, state, and ACO enrollment. Several important patterns emerge.

First, we observe substantial drug-related spending among people with ADRD, particularly among dually eligible beneficiaries and those with multiple chronic conditions. Medicare Part D payments increased markedly with dual-eligibility duration, which may reflect a higher comorbidity burden and medication needs among Medicaid-eligible beneficiaries, as well as a shift of medication-related costs from beneficiaries to Medicare through Medicaid and low-income subsidy protections. These patterns highlight the potential value of integrated care delivery and financing strategies for dually eligible populations,^22^^,^^23^^,^^24^ particularly those with ADRD and multiple chronic conditions who may face elevated risk of fragmented care and complex healthcare needs.^6^^,^^11^^,^^16^

Second, we find pronounced state-level variation in Medicare Part D drug spending, particularly among dually eligible beneficiaries.^21^ Although Medicare Part D cost-sharing rules are largely federally defined, differences in utilization, prescribing patterns, low-income subsidy take-up, and underlying health status likely contribute to this variation.^12^^,^^14^ State-level Medicaid eligibility pathways and enrollment practices may further influence who becomes dually eligible and when, indirectly shaping observed spending patterns.^12^^,^^13^ State Medicaid programs also differ in prescription drug benefit design, including preferred drug lists, prior authorization, copayment policies, and reimbursement methods,^12^^,^^13^ which may influence medication access and prescribing patterns. These findings suggest that geographic variation in Part D spending may reflect differences in medication use, access, and state Medicaid program design rather than cost-sharing policy alone.^12–14^

OOP burden also varies substantially by dual eligibility status. Medicare FFS-only beneficiaries incur approximately $1200-$2000 more in annual OOP drug costs than beneficiaries with full-year dual eligibility. In some states, dual-eligible beneficiaries face minimal OOP costs, whereas FFS-only beneficiaries experience a substantially higher financial burden. These findings highlight the continued vulnerability of Medicare-only beneficiaries to drug-related financial strain and the protective role of Medicaid coverage for dual-eligible individuals.

We also observe wide variation in Medicare Part B drug spending by dual eligibility and state. Part B drug payments are highest among FFS-only beneficiaries and vary by several hundred dollars across enrollment and dual status groups. Although beneficiary OOP costs for Part B drugs are modest overall, state-level differences are substantial. For example, among dually eligible beneficiaries, total Part B payments range from $179 in Vermont to $1446 in Nevada, and OOP costs range from $34 to $362, respectively. These large geographic differences suggest uneven access to provider-administered medications and outpatient clinical infrastructure for people living with ADRD. Such variation has important implications as infusion-based dementia therapies and other provider-administered treatments become more widely available, potentially exacerbating disparities in access and system readiness. Despite strong evidence that ACOs reduce hospitalizations and improve care coordination,^5–7^ our findings are consistent with prior studies showing limited associations between ACO participation and prescription drug spending for both Part D and Part B medications.^10^^,^^15^^,^^25^ This limited impact likely reflects structural constraints, including the exclusion of Part D spending from direct ACO financial accountability and limited incentives tied to pharmacy-related outcomes.^15^^,^^25^^,^^26^ Although ACOs engage with Part B services through outpatient and physician-administered care, our results suggest that their influence on medication spending remains modest, particularly among people with ADRD who have complex care needs.

These findings have several practical implications for clinicians and policymakers. First, medication affordability and regimen complexity among Medicare-only beneficiaries with ADRD should be treated as a routine clinical risk factor, analogous to other social and financial barriers, and should trigger targeted supports such as regimen simplification and pharmacy consultation. Second, state-level variation in Part B drug spending suggests a need to strengthen outpatient clinical capacity to deliver provider-administered therapies, including infusion services and related monitoring infrastructure, in ways that reduce geographic disparities. Third, value-based care models may require stronger pharmacy integration and aligned incentives across Medicare Parts B and D and Medicaid to support high-quality medication management for people with ADRD.^8^^,^^26^^,^^27^

This study has several limitations. First, our analysis relies on Medicare FFS claims and captures only Medicare-covered spending. We do not observe Medicaid payments or non-covered services, which may lead to underestimation of total medication costs, particularly for dually eligible beneficiaries. Second, although we adjust for demographic characteristics and comorbidity burden, we do not incorporate CMS risk adjustment measures such as Hierarchical Condition Categories or Prescription Drug HCC scores, and residual confounding related to health status, geography, or ACO attribution may remain. Third, our analysis focuses on beneficiaries attributed to MSSP ACOs and may not generalize to Medicare Advantage enrollees or other ACO models. Fourth, we do not directly examine low-income subsidy enrollment, which is closely related to but distinct from dual eligibility and may independently influence Part D cost sharing and utilization. We also do not observe detailed drug benefit design or supplemental coverage features, including Part D formularies, deductibles, and prior authorization, which may influence medication use and OOP spending. In addition, we lack direct measures of socioeconomic status, such as income, and therefore cannot account for “near-poor” beneficiaries who may not qualify for Medicaid even though they experience substantial OOP medication burden. Finally, we lack measures of ADRD severity, which may affect treatment intensity and medication spending.

Recent federal initiatives under the Inflation Reduction Act (IRA), including Medicare drug price negotiations and the $2000 annual Part D OOP cap,^28^ represent important steps toward improving medication affordability for older adults with complex conditions, such as ADRD. Prior studies have projected substantial reductions in OOP prescription drug spending under the IRA, particularly among beneficiaries with high medication use and chronic conditions.^29^^,^^30^ Our findings provide important policy context by demonstrating substantial variation in Medicare and beneficiary drug spending across dual eligibility, ACO enrollment, and states among older adults with ADRD. The results also suggest that medication-related financial burden extends beyond Part D prescription drugs to include Part B provider-administered therapies, which are less directly affected by the IRA’s Part D redesign and may become increasingly important as coverage of disease-modifying therapies for ADRD expands. These findings highlight the importance of integrating medication management, care coordination, and aligned incentives across Medicare Part B, Part D, and Medicaid to support equitable and cost-effective care for people living with ADRD.

## Supplementary material

Supplementary material is available at *Innovation in Aging* online.

## Supplementary Material

igag085_Supplementary_Data

## Data Availability

The University of Maryland Institutional Review Board approved the data used in this study. The study relied on restricted CMS Medicare claims data, which are not publicly available. Access to these data may be requested from the Research Data Assistance Center (ResDAC) by qualified researchers with appropriate data use agreements and approvals. The study was not preregistered.
