# Supplementary material for: Variation in Medicare Part D and Part B drug spending among older adults with Alzheimer’s disease and related dementias
Source: Innov Aging. 2026 Aug 3;10(10):igag085. doi: 10.1093/geroni/igag085 (PMC13602412; doi:10.1093/geroni/igag085)
Supplement: Supplementary file 1 [file igag085_supplementary_data.zip › innage_suppl_Jang_&_Chen.pdf]

Supplementary Table 1. Examples of Medication Categories Covered under Medicare Part D versus Part B among Beneficiaries with ADRD

**Coverage**	**Examples of medication categories**
Part D prescription drugs	Donepezil, rivastigmine, galantamine, memantine; antidepressants; antipsychotics; antihypertensives; diabetes medications; anticoagulants
Part B provider-administered drugs	Aducanumab, with limited Medicare coverage/use in 2022; injectable or infused drugs administered in physician offices or outpatient settings for comorbid conditions, such as biologics, chemotherapy agents, antiemetics, osteoporosis injections, or other clinician-administered therapies

Notes: Examples are illustrative and do not represent the most frequently used medications in the study sample. Lecanemab and donanemab were not available during the 2022 study period.

Supplemental Table 2. Two-part Model on Part D beneficiary OOP spending among FFS beneficiaries with ADRD

	**FFS only**				**Dually eligible for 1 to 11 months**				**Dually eligible for 12 months**			
**Part 1. Logit**	OR	95% CI		*p*	OR	95% CI		*p*	OR	95% CI		*p*
ACO (ref: Never/Partial)												
Full-year enrollment	0.93	0.87	1.00	0.043	1.28	1.19	1.38	<0.001	1.55	1.52	1.58	<0.001
Gender (ref: Male)												
Female	1.08	1.01	1.16	0.028	1.02	0.95	1.08	0.623	0.98	0.97	1.00	0.040
Age (ref: 65-74)												
75-84	0.87	0.79	0.96	0.008	0.91	0.84	0.98	0.011	0.90	0.88	0.91	<0.001
85 or older	0.73	0.66	0.80	<0.001	0.78	0.72	0.84	<0.001	0.67	0.66	0.69	<0.001
Race/Ethnicity												
Black	0.70	0.61	0.81	<0.001	0.68	0.61	0.75	<0.001	1.16	1.13	1.18	<0.001
Hispanic	0.89	0.72	1.09	0.261	1.40	1.23	1.59	<0.001	2.34	2.28	2.39	<0.001
Other	0.68	0.58	0.80	<0.001	1.13	1.00	1.29	0.049	2.04	1.99	2.09	<0.001
Part D coverage												
12-month enrollment	2.57	2.20	3.00	<0.001	13.26	12.29	14.30	0.000	0.79	0.72	0.87	<0.001
No. of cc (ref: 0 or 1)												
2 to 4	1.85	1.69	2.03	<0.001	0.97	0.87	1.08	0.616	0.88	0.84	0.91	<0.001
5 or more	4.69	4.19	5.25	<0.001	1.01	0.90	1.12	0.914	0.84	0.81	0.88	<0.001
**Part 2. GLM**	MR	95% CI		*p*	MR	95% CI		*p*	MR	95% CI		*p*
ACO (ref: Never/Partial)												
Full-year enrollment	0.97	0.96	0.98	<0.001	1.08	1.01	1.15	0.024	1.00	0.98	1.02	0.882
Gender (ref: Male)												
Female	0.87	0.86	0.88	<0.001	0.89	0.84	0.95	<0.001	0.96	0.94	0.98	<0.001
Age (ref: 65-74)												
75-84	0.94	0.93	0.95	<0.001	1.21	1.12	1.31	<0.001	0.95	0.93	0.98	<0.001

85 or older	0.85	0.83	0.86	<0.001	1.06	0.98	1.14	0.132	0.86	0.84	0.88	<0.001
Race/Ethnicity												
Black	0.95	0.93	0.97	<0.001	0.68	0.61	0.75	<0.001	0.78	0.75	0.80	<0.001
Hispanic	0.86	0.83	0.89	<0.001	0.61	0.54	0.69	<0.001	0.70	0.69	0.72	<0.001
Other	1.01	0.98	1.03	0.701	0.62	0.55	0.71	<0.001	0.75	0.73	0.77	<0.001
Part D coverage												
12-month enrollment	1.70	1.64	1.77	<0.001	5.10	4.71	5.52	<0.001	1.25	1.13	1.39	<0.001
No. of cc (ref: 0 or 1)												
2 to 4	1.56	1.53	1.59	<0.001	1.83	1.63	2.05	<0.001	1.78	1.71	1.85	<0.001
5 or more	2.36	2.31	2.41	<0.001	2.67	2.38	3.00	<0.001	2.69	2.59	2.81	<0.001
	Part 1 N=647,313; Part 2 N=643,865				Part 1 N=30,313; Part 2 N=19,456				Part 1 N=340,173; Part 2 N=125,559			

Notes: Two-part models were used to examine Part D beneficiary payments among community-dwelling FFS beneficiaries with ADRD. Part 1 estimated the odds of any Part D drug OOP spending using logistic regression, with coefficients reported as odds ratios. Part 2 estimated Part D OOP spending among beneficiaries with positive drug spending using generalized linear models with a log link and gamma distribution, with exponentiated coefficients interpreted as mean ratios. States were controlled but omitted due to limited space. Percentages of zero-OOP spending are 0.5% for FFS only, 35.8% for Dually eligible 1-11 months, and 63.1% for Dually eligible 12 months.

	**FFS only**				**Dually eligible for 1 to 11 months**				**Dually eligible for 12 months**			
ACO enrollment	ME	95% CI		*p*	ME	95% CI		*p*	ME	95% CI		*p*
Never/Partial	1,213.81	1,205.60	1,222.02	<0.001	302.79	291.68	313.90	<0.001	31.20	30.82	31.59	<0.001
Full-year enrollment	1,181.05	1,170.95	1,191.16		337.78	318.97	356.58		39.32	38.49	40.15	

Notes: Adjusted predicted drug spending was estimated using predictive margins after two-part models. *p*-values were obtained from post-estimation Wald tests comparing ACO enrollees and non-ACO beneficiaries. *Abbreviations*: ME, marginal effects; 95% CI, confidence intervals.

Supplemental Table 3. Two-part Model on Part B drug beneficiary OOP spending among FFS beneficiaries with ADRD

	FFS only				Dually eligible for 1 to 11 months				Dually eligible for 12 months			
**Part 1. Logit**	OR	95% CI		*p*	OR	95% CI		*p*	OR	95% CI		*p*
ACO (ref: Never/Partial)												
Full-year enrollment	0.89	0.88	0.90	<0.001	0.84	0.78	0.89	<0.001	0.79	0.77	0.81	<0.001
Gender (ref: Male)												
Female	0.95	0.94	0.96	<0.001	0.98	0.92	1.04	0.482	1.00	0.98	1.02	0.823
Age (ref: 65-74)												
75-84	0.84	0.83	0.86	<0.001	0.87	0.81	0.94	<0.001	0.95	0.93	0.97	<0.001
85 or older	0.74	0.73	0.75	<0.001	0.92	0.85	0.99	0.022	0.91	0.89	0.93	<0.001
Race/Ethnicity												
Black	0.89	0.87	0.91	<0.001	1.02	0.93	1.13	0.661	0.88	0.85	0.90	<0.001
Hispanic	1.00	0.97	1.03	0.899	1.07	0.94	1.21	0.325	0.93	0.91	0.96	<0.001
Other	0.81	0.79	0.83	<0.001	0.84	0.73	0.95	0.007	0.67	0.65	0.68	<0.001
No. of cc (ref: 0 or 1)												
2 to 4	2.01	1.97	2.05	<0.001	1.84	1.64	2.08	<0.001	1.90	1.81	1.98	<0.001
5 or more	4.55	4.46	4.65	<0.001	3.90	3.46	4.40	<0.001	4.21	4.02	4.40	<0.001
**Part 2. GLM**	MR	95% CI		*p*	MR	95% CI		*p*	MR	95% CI		*p*
ACO (ref: Never/Partial)												
Full-year enrollment	1.03	0.99	1.07	0.147	1.36	1.10	1.68	0.004	1.09	0.97	1.23	0.149
Gender (ref: Male)												
Female	0.86	0.83	0.90	<0.001	1.08	0.89	1.31	0.441	0.93	0.83	1.03	0.160
Age (ref: 65-74)												
75-84	0.90	0.85	0.95	<0.001	0.79	0.62	1.00	0.052	1.00	0.88	1.13	0.989
85 or older	0.86	0.82	0.91	<0.001	0.82	0.65	1.04	0.107	0.93	0.82	1.05	0.242
Race/Ethnicity												

Black	1.04	0.96	1.14	0.340	1.33	0.95	1.85	0.092	1.33	1.14	1.54	<0.001
Hispanic	0.84	0.75	0.94	0.003	0.76	0.50	1.16	0.204	0.90	0.78	1.04	0.167
Other	0.90	0.81	1.01	0.070	0.70	0.44	1.09	0.114	0.88	0.76	1.03	0.116
No. of cc (ref: 0 or 1)												
2 to 4	1.05	0.95	1.17	0.342	1.19	0.73	1.92	0.485	0.87	0.63	1.21	0.412
5 or more	1.29	1.16	1.43	<0.001	1.52	0.94	2.45	0.087	1.16	0.84	1.60	0.380
	Part 1 N=738,296; Part 2 N=397,318				Part 1 N=22,467; Part 2 N=13,332				Part 1 N=236,986; Part 2 N=146,928			

Notes: Two-part models were used to examine Part B drug beneficiary payments among community-dwelling FFS beneficiaries with ADRD. Part 1 estimated the odds of any Part B drug OOP spending using logistic regression, with coefficients reported as odds ratios. Part 2 estimated Part B OOP spending among beneficiaries with positive drug spending using generalized linear models with a log link and gamma distribution, with exponentiated coefficients interpreted as mean ratios. States were controlled but omitted due to limited space. Percentages of zero-OOP spending are 46.2% for FFS only, 40.7% for Dually eligible 1-11 months, and 38.0% for Dually eligible 12 months.

	**FFS only**				**Dually eligible for 1 to 11 months**				**Dually eligible for 12 months**			
ACO enrollment	ME	95% CI		*p*	ME	95% CI		*p*	ME	95% CI		*p*
Never/Partial	269.27	262.62	275.92	0.411	136.33	121.54	151.12	0.036	173.11	163.52	182.70	0.941
Full-year enrollment	264.67	255.93	273.41		173.41	141.34	205.49		173.90	154.53	193.27	

Notes: Adjusted predicted drug spending was estimated using predictive margins after two-part models. *p*-values were obtained from post-estimation Wald tests comparing ACO enrollees and non-ACO beneficiaries. *Abbreviations*: ME, marginal effects; 95% CI, confidence intervals.

Supplemental Table 4. Two-part Model on Part D drug beneficiary OOP spending among ADRD beneficiaries with 5 or more chronic conditions

	FFS only				Dual 1-11 months				Dual 12 months			
**Part 1. Logit**	OR	95% CI		*p*	OR	95% CI		*p*	OR	95% CI		*p*
ACO (ref: Never/Partial)												
Full-year enrollment	1.04	0.89	1.23	0.596	1.37	1.22	1.54	0.000	1.62	1.58	1.66	0.000
Gender (ref: Male)												
Female	1.11	0.95	1.30	0.185	0.99	0.89	1.10	0.848	0.99	0.96	1.01	0.263
Age (ref: 65-74)												
75-84	0.82	0.64	1.04	0.096	0.90	0.79	1.01	0.084	0.84	0.82	0.87	0.000
85 or older	0.65	0.51	0.83	0.000	0.77	0.68	0.87	0.000	0.64	0.62	0.65	0.000
Race/Ethnicity												
Black	0.54	0.40	0.71	0.000	0.55	0.47	0.64	0.000	1.02	0.99	1.06	0.191
Hispanic	0.62	0.42	0.93	0.020	1.35	1.07	1.69	0.010	1.91	1.85	1.97	0.000
Other	0.64	0.43	0.94	0.023	1.08	0.87	1.35	0.470	1.62	1.56	1.68	0.000
Part D coverage												
12-month enrollment	4.83	3.65	6.39	0.000	16.31	14.42	18.46	0.000	0.81	0.70	0.95	0.007
**Part 2. GLM**	MR	95% CI		*p*	MR	95% CI		*p*	MR	95% CI		*p*
ACO (ref: Never/Partial)												
Full-year enrollment	0.97	0.96	0.98	0.000	1.05	0.97	1.13	0.197	0.97	0.94	0.99	0.003
Gender (ref: Male)												
Female	0.89	0.88	0.90	0.000	0.87	0.80	0.94	0.000	0.99	0.97	1.01	0.357
Age (ref: 65-74)												
75-84	0.92	0.91	0.94	0.000	1.19	1.09	1.31	0.000	0.96	0.94	0.99	0.003
85 or older	0.80	0.79	0.82	0.000	1.02	0.92	1.12	0.739	0.87	0.85	0.90	0.000
Race/Ethnicity												
Black	1.00	0.97	1.03	0.956	0.79	0.70	0.90	0.000	0.80	0.77	0.82	0.000

Hispanic	0.88	0.84	0.91	0.000	0.65	0.56	0.76	0.000	0.71	0.69	0.73	0.000
Other	1.01	0.97	1.05	0.552	0.61	0.51	0.72	0.000	0.77	0.75	0.79	0.000
Part D coverage												
12-month enrollment	1.72	1.64	1.81	0.000	4.62	4.15	5.15	0.000	1.10	0.97	1.25	0.122
	Part 1 N=262,982; Part 2 N=261,468				Part 1 N=13,007; Part 2 N=8,698				Part 1 N=170,263; Part 2 N=63,554			

Notes: Two-part models were used to examine Part D beneficiary payments among community-dwelling FFS beneficiaries with ADRD and five or more chronic conditions. Part 1 estimated the odds of any Part D drug OOP spending using logistic regression, with coefficients reported as odds ratios. Part 2 estimated Part D OOP spending among beneficiaries with positive drug spending using generalized linear models with a log link and gamma distribution, with exponentiated coefficients interpreted as mean ratios. States were controlled but omitted due to limited space. Percentages of zero-OOP spending are 0.6% for FFS only, 33.1% for Dually eligible 1-11 months, and 62.7% for Dually eligible 12 months.

	**FFS only**				**Dually eligible for 1 to 11 months**				**Dually eligible for 12 months**			
ACO enrollment	ME	95% CI		*p*	ME	95% CI		*p*	ME	95% CI		*p*
Never/Partial	1,555.22	1,541.75	1,568.69	<0.001	404.83	387.23	422.43	0.026	38.39	37.87	38.92	<0.001
Full-year enrollment	1,508.54	1,492.35	1,524.74		442.80	413.74	471.85		47.40	46.29	48.50	

Notes: Adjusted predicted drug spending was estimated using predictive margins after two-part models. *p*-values were obtained from post-estimation Wald tests comparing ACO enrollees and non-ACO beneficiaries. *Abbreviations*: ME, marginal effects; 95% CI, confidence intervals.

Supplemental Table 5. Two-part Model on Part B drug beneficiary OOP spending among ADRD beneficiaries with 5 or more chronic conditions

	FFS only				Dually eligible for 1 to 11 months				Dually-eligible for 12 months			
**Part 1. Logit**	OR	95% CI		*p*	OR	95% CI		*p*	OR	95% CI		*p*
ACO (ref: Never/Partial)												
Full-year enrollment	0.91	0.90	0.92	0.000	0.89	0.81	0.98	0.016	0.81	0.78	0.83	0.000
Gender (ref: Male)												
Female	0.98	0.96	0.99	0.002	1.09	0.99	1.20	0.083	1.03	1.00	1.06	0.054
Age (ref: 65-74)												
75-84	0.81	0.80	0.83	0.000	0.80	0.72	0.90	0.000	0.92	0.89	0.95	0.000
85 or older	0.66	0.64	0.67	0.000	0.79	0.70	0.89	0.000	0.85	0.82	0.88	0.000
Race/Ethnicity												
Black	0.94	0.91	0.97	0.001	1.13	0.97	1.32	0.115	0.86	0.83	0.89	0.000
Hispanic	1.04	0.99	1.09	0.118	1.01	0.83	1.24	0.891	0.94	0.90	0.97	0.001
Other	0.83	0.80	0.87	0.000	0.84	0.68	1.04	0.114	0.69	0.66	0.72	0.000
**Part 2. GLM**	MR	95% CI		*p*	MR	95% CI		*p*	MR	95% CI		*p*
ACO (ref: Never/Partial)												
Full-year enrollment	1.03	0.98	1.08	0.244	1.24	0.96	1.59	0.097	1.11	0.94	1.30	0.209
Gender (ref: Male)												
Female	0.86	0.82	0.90	0.000	0.97	0.76	1.23	0.804	0.92	0.80	1.06	0.269
Age (ref: 65-74)												
75-84	0.86	0.80	0.92	0.000	0.74	0.55	0.98	0.036	0.95	0.81	1.13	0.575
85 or older	0.75	0.70	0.81	0.000	0.77	0.58	1.03	0.082	0.83	0.70	0.98	0.031
Race/Ethnicity												
Black	0.96	0.87	1.07	0.490	1.25	0.84	1.87	0.275	1.32	1.07	1.61	0.008
Hispanic	0.90	0.79	1.03	0.137	0.57	0.35	0.93	0.026	0.82	0.68	1.00	0.045

Other	0.88	0.76	1.01	0.071	0.91	0.51	1.63	0.761	0.79	0.64	0.97	0.024
	Part 1 N=314,551; Part 2 N=209,579				Part 1 N=10,523; Part 2 N=7,329				Part 1 N=129,098; Part 2 N=92,128			

Notes: Two-part models were used to examine Part B drug beneficiary payments among community-dwelling FFS beneficiaries with ADRD and five or more chronic conditions. Part 1 estimated the odds of any Part B drug OOP spending using logistic regression, with coefficients reported as odds ratios. Part 2 estimated Part B drug OOP spending among beneficiaries with positive drug spending using generalized linear models with a log link and gamma distribution, with exponentiated coefficients interpreted as mean ratios. States were controlled but omitted due to limited space. Percentages of zero-OOP spending are 33.4% for FFS only, 30.4% for Dually eligible 1-11 months, and 28.6% for Dually eligible 12 months.

	**FFS only**				**Dually eligible for 1 to 11 months**				**Dually eligible for 12 months**			
ACO enrollment	ME	95% CI		*p*	ME	95% CI		*p*	ME	95% CI		*p*
Never/Partial	365.49	354.31	376.66	0.993	175.27	152.57	197.97	0.189	222.42	205.83	239.00	0.624
Full-year enrollment	365.41	350.87	379.95		209.31	163.32	255.30		231.76	196.81	266.71	

Notes: Adjusted predicted drug spending was estimated using predictive margins after two-part models. *p*-values were obtained from post-estimation Wald tests comparing ACO enrollees and non-ACO beneficiaries. *Abbreviations*: ME, marginal effects; 95% CI, confidence intervals.

Supplementary Table 6. Sample characteristics among community-living FFS Medicare beneficiaries with ADRD by ACO enrollment and Medicare-Medicaid dual eligibility coverage levels

	**FFS only**			**Dually eligible 1 to 11 months**			**Dually eligible for 12 months**		
	**Non-ACO**	**ACO**		**Non-ACO**	**ACO**		**Non-ACO**	**ACO**	
	634,171 (64.2)	354,186 (35.8)	*p*	26,831 (76.8)	8,100 (23.2)	*p*	273,849 (78.4)	75,559 (21.6)	*p*
Age group			<0.001			<0.001			<0.001
65-74	107,412 (16.9)	55,918 (15.8)		7,645 (28.5)	1,451 (17.9)		69,007 (25.2)	16,688 (22.1)	
75-84	258,567 (40.8)	154,769 (43.7)		9,024 (33.6)	2,951 (36.4)		96,987 (35.4)	26,895 (35.6)	
85 or older	268,192 (42.3)	143,499 (40.5)		10,162 (37.9)	3,698 (45.7)		107,855 (39.4)	31,976 (42.3)	
Sex			<0.001			<0.001			<0.001
Male	251,354 (39.6)	144,248 (40.7)		8,788 (32.8)	2,124 (26.2)		82,937 (30.3)	20,836 (27.6)	
Female	382,817 (60.4)	209,938 (59.3)		18,043 (67.3)	5,976 (73.8)		190,912 (69.7)	54,723 (72.4)	
Race/Ethnicity			<0.001			<0.001			<0.001
NH White	546,054 (86.1)	313,258 (88.4)		20,005 (74.6)	6,738 (83.2)		152,012 (55.5)	45,763 (60.6)	
Black	40,025 (6.3)	19,821 (5.6)		3,222 (12.0)	742 (9.2)		41,313 (15.1)	10,530 (13.9)	
Hispanic	21,423 (3.4)	9,598 (2.7)		1,864 (7.0)	379 (4.7)		41,513 (15.2)	10,972 (14.5)	
Other	26,669 (4.2)	11,509 (3.3)		1,740 (6.5)	241 (3.0)		39,011 (14.3)	8,294 (11.0)	
Part D coverage			<0.001			<0.001			<0.001
Full-year	399,447 (63.0)	244,573 (69.1)		16,779 (62.5)	6,245 (77.1)		269,491 (98.4)	74,532 (98.6)	
No. of comorbidities			<0.001			<0.001			0.501
0 to 4	393,396 (62.0)	206,910 (58.4)		16,184 (60.3)	4,369 (53.9)		138,273 (50.5)	38,256 (50.6)	
5 or more	240,775 (38.0)	147,276 (41.6)		10,647 (39.7)	3,731 (46.1)		135,576 (49.5)	37,303 (49.4)	

Notes: Values are presented as number (percentage). The study sample included community-living Medicare fee-for-service (FFS) beneficiaries with Alzheimer's disease and related dementias (ADRD) who were continuously enrolled in FFS in 2022. ACO enrollment was defined as full-year enrollment in a Medicare Shared Savings Program Accountable Care Organization (ACO); beneficiaries with no or partial-year ACO enrollment were classified as non-ACO. Dual eligibility was categorized as no months of Medicare-Medicaid dual eligibility, 1–11 months of dual eligibility, or 12 months of dual eligibility during the year. *p*-values compare characteristics between ACO and non-ACO beneficiaries within each dual-eligibility group and were calculated using chi-squared tests.

Supplemental Table 7. GLM results on Part D drug Medicare payments among FFS beneficiaries with ADRD

	FFS only				Dually eligible for 1 to 11 months				Dually-eligible for 12 months			
	MR	95% CI		*p*	MR	95% CI		*p*	MR	95% CI		*p*
ACO (ref: Never/Partial)												
Full-year enrollment	1.02	0.99	1.04	0.166	1.02	0.95	1.09	0.606	1.04	1.02	1.06	<0.001
Gender (ref: Male)												
Female	0.73	0.71	0.75	<0.001	0.87	0.82	0.93	<0.001	0.89	0.87	0.90	<0.001
Age (ref: 65-74)												
75-84	0.79	0.76	0.81	<0.001	0.58	0.54	0.63	<0.001	0.76	0.74	0.77	<0.001
85 or older	0.57	0.55	0.59	<0.001	0.44	0.40	0.47	<0.001	0.54	0.53	0.55	<0.001
Race/Ethnicity												
Black	1.04	0.99	1.10	0.126	1.28	1.16	1.42	<0.001	0.86	0.84	0.88	<0.001
Hispanic	0.97	0.91	1.04	0.448	1.23	1.08	1.40	0.002	0.94	0.92	0.96	<0.001
Other	1.12	1.05	1.19	0.001	1.28	1.12	1.47	<0.001	1.08	1.05	1.10	<0.001
Part D coverage												
12-month enrollment	2.05	1.89	2.23	<0.001	1.90	1.76	2.06	<0.001	1.94	1.78	2.11	<0.001
No. of cc (ref: 0 or 1)												
2 to 4	1.78	1.70	1.86	<0.001	1.68	1.50	1.89	<0.001	1.79	1.73	1.85	<0.001
5 or more	3.14	3.00	3.29	<0.001	2.96	2.63	3.33	<0.001	3.39	3.28	3.51	<0.001

Notes: Generalized linear models with a log link and gamma distribution were used to examine Part D Medicare payments, with exponentiated coefficients interpreted as mean ratios. States were controlled but omitted due to limited space. N for FFS only=647,313; N for 1-11 months of dual=30,313; N for 12 months of dual=340,173. *Abbreviations*: ACO, accountable care organization; No. of cc, number of chronic conditions.

	FFS only				Dually eligible for 1 to 11 months				Dually eligible for 12 months			
ACO enrollment	ME	95% CI		*p*	ME	95% CI		*p*	ME	95% CI		*p*
Never/Partial	2,930.50	2,884.57	2,976.43	0.167	4,882.75	4,693.51	5,071.99	0.607	8,964.90	8,888.20	9,041.61	<0.001
Full-year enrollment	2,981.55	2,923.19	3,039.92		4,974.55	4,654.46	5,294.63		9,303.81	9,156.15	9,451.47	

Notes: Adjusted predicted drug spending were estimated using predictive margins after generalized linear models. *p*-values were obtained from post-estimation Wald tests comparing ACO enrollees and non-ACO beneficiaries. *Abbreviations*: ME, marginal effects; 95% CI, confidence intervals.

Supplemental Table 8. GLM results on Part D drug beneficiary OOP spending among FFS beneficiaries with ADRD

	**FFS only**				**Dually eligible for 1 to 11 months**				**Dually-eligible for 12 months**			
	MR	95% CI		*p*	MR	95% CI		*p*	MR	95% CI		*p*
ACO (ref: Never/Partial)												
Full-year enrollment	0.97	0.96	0.98	<0.001	1.19	1.09	1.30	<0.001	1.35	1.30	1.41	<0.001
Gender (ref: Male)												
Female	0.87	0.86	0.88	<0.001	0.92	0.85	1.00	0.047	0.94	0.90	0.98	0.001
Age (ref: 65-74)												
75-84	0.94	0.92	0.95	<0.001	1.28	1.16	1.41	<0.001	0.87	0.83	0.91	<0.001
85 or older	0.85	0.83	0.86	<0.001	1.06	0.96	1.17	0.276	0.63	0.60	0.66	<0.001
Race/Ethnicity												
Black	0.95	0.93	0.97	<0.001	0.55	0.49	0.62	<0.001	0.86	0.81	0.90	<0.001
Hispanic	0.86	0.83	0.89	<0.001	0.70	0.59	0.82	<0.001	1.14	1.08	1.20	<0.001
Other	1.00	0.97	1.03	0.829	0.63	0.53	0.75	<0.001	1.06	1.00	1.12	0.062
Part D coverage												
12-month enrollment	1.72	1.66	1.78	<0.001	9.64	8.80	10.56	<0.001	0.91	0.74	1.11	0.346
No. of cc (ref: 0 or 1)												
2 to 4	1.57	1.53	1.60	<0.001	1.75	1.51	2.02	<0.001	1.62	1.50	1.76	<0.001
5 or more	2.38	2.33	2.43	<0.001	2.58	2.23	2.98	<0.001	2.35	2.17	2.55	<0.001

Notes: Generalized linear models with a log link and gamma distribution were used to examine Part D beneficiary payments, with exponentiated coefficients interpreted as mean ratios. States were controlled but omitted due to limited space. N for FFS only=647,313; N for 1-11 months of dual=30,313; N for 12 months of dual=340,173.
Abbreviations: ACO, accountable care organization; No. of cc, number of chronic conditions.

	FFS only				Dually eligible for 1 to 11 months				Dually eligible for 12 months			
ACO enrollment	ME	95% CI		*p*	ME	95% CI		*p*	ME	95% CI		*p*
Never/Partial	1,213.87	1,205.65	1,222.08	<0.001	290.46	275.09	305.84	<0.001	31.02	30.35	31.69	<0.001
Full-year enrollment	1,181.04	1,170.93	1,191.15		346.59	318.97	374.21		41.98	40.29	43.66	

Notes: Adjusted predicted drug spending were estimated using predictive margins after generalized linear models. *p*-values were obtained from post-estimation Wald tests comparing ACO enrollees and non-ACO beneficiaries. *Abbreviations*: ME, marginal effects; 95% CI, confidence intervals.

Supplemental Table 9. GLM results on Part B drug Medicare payments among FFS beneficiaries with ADRD

	FFS only				Dually eligible for 1 to 11 months				Dually-eligible for 12 months			
	MR	95% CI		*p*	MR	95% CI		*p*	MR	95% CI		*p*
ACO (ref: Never/Partial)												
Full-year enrollment	1.00	0.96	1.04	0.894	1.27	1.06	1.53	0.009	1.03	0.93	1.14	0.605
Gender (ref: Male)												
Female	0.85	0.82	0.88	<0.001	1.07	0.90	1.28	0.441	0.93	0.85	1.03	0.150
Age (ref: 65-74)												
75-84	0.85	0.81	0.90	<0.001	0.77	0.62	0.95	0.015	0.98	0.88	1.10	0.783
85 or older	0.79	0.75	0.83	<0.001	0.79	0.64	0.98	0.030	0.90	0.81	1.01	0.068
Race/Ethnicity												
Black	0.98	0.90	1.06	0.609	1.26	0.93	1.70	0.135	1.24	1.09	1.42	0.002
Hispanic	0.84	0.75	0.93	0.001	0.87	0.60	1.28	0.486	0.91	0.80	1.04	0.162
Other	0.84	0.76	0.92	<0.001	0.68	0.46	0.99	0.043	0.82	0.72	0.93	0.003
No. of cc (ref: 0 or 1)												
2 to 4	1.48	1.37	1.59	<0.001	1.47	1.05	2.07	0.027	1.19	0.95	1.49	0.127
5 or more	2.42	2.25	2.60	<0.001	2.34	1.66	3.29	0.000	2.00	1.60	2.50	<0.001

Notes: Generalized linear models with a log link and gamma distribution were used to examine Part B drug Medicare payments, with exponentiated coefficients interpreted as mean ratios. States were controlled but omitted due to limited space. N for FFS only=738,296; N for 1-11 months of dual=22,467; N for 12 months of dual=236,986. *Abbreviations*: ACO, accountable care organization; No. of cc, number of chronic conditions.

	FFS only				Dually eligible for 1 to 11 months				Dually eligible for 12 months			
ACO enrollment	ME	95% CI		*p*	ME	95% CI		*p*	ME	95% CI		*p*
Never/Partial	1,171.94	1,144.43	1,199.45	0.894	598.86	539.35	658.36	0.015	743.33	706.14	780.52	0.608
Full-year enrollment	1,168.94	1,133.48	1,204.41		762.95	640.41	885.49		764.00	691.02	836.98	

Notes: Adjusted predicted drug spending were estimated using predictive margins after generalized linear models. *p*-values were obtained from post-estimation Wald tests comparing ACO enrollees and non-ACO beneficiaries. *Abbreviations*: ME, marginal effects; 95% CI, confidence intervals.

Supplemental Table 10. GLM results on Part B drug beneficiary OOP spending among FFS beneficiaries with ADRD

	FFS only				Dually eligible for 1 to 11 months				Dually-eligible for 12 months			
	MR	95% CI		*p*	MR	95% CI		*p*	MR	95% CI		*p*
ACO (ref: Never/Partial)												
Full-year enrollment	0.98	0.94	1.02	0.417	1.25	1.02	1.54	0.031	1.00	0.89	1.11	0.932
Gender (ref: Male)												
Female	0.84	0.81	0.87	<0.001	1.06	0.87	1.30	0.533	0.93	0.84	1.02	0.139
Age (ref: 65-74)												
75-84	0.84	0.79	0.89	<0.001	0.76	0.59	0.96	0.024	1.00	0.88	1.12	0.941
85 or older	0.79	0.74	0.83	<0.001	0.82	0.64	1.04	0.103	0.92	0.82	1.04	0.196
Race/Ethnicity												
Black	0.98	0.89	1.07	0.651	1.23	0.87	1.73	0.239	1.25	1.08	1.44	0.002
Hispanic	0.83	0.74	0.94	0.002	0.84	0.54	1.30	0.423	0.89	0.77	1.03	0.111
Other	0.81	0.72	0.90	<0.001	0.58	0.38	0.90	0.014	0.77	0.67	0.88	<0.001
No. of cc (ref: 0 or 1)												
2 to 4	1.62	1.49	1.75	<0.001	1.78	1.20	2.64	0.004	1.31	1.03	1.66	0.029
5 or more	2.81	2.59	3.05	<0.001	3.06	2.06	4.55	<0.001	2.35	1.85	2.99	<0.001

Notes: Generalized linear models with a log link and gamma distribution were used to examine Part B drug beneficiary payments, with exponentiated coefficients interpreted as mean ratios. States were controlled but omitted due to limited space. N for FFS only=738,296; N for 1-11 months of dual=22,467; N for 12 months of dual=236,986. *Abbreviations*: ACO, accountable care organization; No. of cc, number of chronic conditions.

	FFS only				Dually eligible for 1 to 11 months				Dually eligible for 12 months			
ACO enrollment	ME	95% CI		*p*	ME	95% CI		*p*	ME	95% CI		*p*
Never/Partial	269.38	262.27	276.49	0.416	138.38	122.34	154.42	0.043	172.92	163.55	182.29	0.931
Full-year enrollment	264.75	255.74	273.75		173.45	141.89	205.00		172.08	154.42	189.73	

Notes: Adjusted predicted drug spending were estimated using predictive margins after generalized linear models. *p*-values were obtained from post-estimation Wald tests comparing ACO enrollees and non-ACO beneficiaries. *Abbreviations*: ME, marginal effects; 95% CI, confidence intervals.

Supplemental Table 11. GLM results on Part D drug Medicare payments among FFS beneficiaries with ADRD and five or more chronic conditions

	**FFS only**				**Dually eligible for 1 to 11 months**				**Dually-eligible for 12 months**			
	MR	95% CI		*p*	MR	95% CI		*p*	MR	95% CI		*p*
ACO (ref: Never/Partial)												
Full-year enrollment	1.03	1.00	1.06	0.100	0.98	0.90	1.07	0.675	1.03	1.01	1.05	0.002
Gender (ref: Male)												
Female	0.79	0.77	0.81	<0.001	0.89	0.82	0.96	0.005	0.92	0.91	0.94	<0.001
Age (ref: 65-74)												
75-84	0.77	0.74	0.80	<0.001	0.62	0.56	0.69	<0.001	0.81	0.79	0.83	<0.001
85 or older	0.55	0.53	0.57	<0.001	0.43	0.39	0.48	<0.001	0.59	0.57	0.60	<0.001
Race/Ethnicity												
Black	1.05	0.98	1.12	0.145	1.13	1.00	1.28	0.054	0.82	0.80	0.84	<0.001
Hispanic	1.05	0.96	1.14	0.284	1.06	0.90	1.26	0.477	0.91	0.89	0.93	<0.001
Other	1.21	1.11	1.31	<0.001	1.27	1.06	1.53	0.009	1.06	1.03	1.08	<0.001
Part D coverage												
12-month enrollment	2.14	1.93	2.37	<0.001	1.97	1.79	2.18	0.000	1.88	1.69	2.11	<0.001

Notes: Generalized linear models with a log link and gamma distribution were used to examine Part D Medicare payments, with exponentiated coefficients interpreted as mean ratios. States were controlled but omitted due to limited space. N for FFS only=263,658; N for 1-11 months of dual=13,007; N for 12 months of dual=170,263. *Abbreviations*: ACO, accountable care organization.

	**FFS only**				**Dually eligible for 1 to 11 months**				**Dually eligible for 12 months**			
ACO enrollment	ME	95% CI		*p*	ME	95% CI		*p*	ME	95% CI		*p*
Never/Partial	4,025.34	3,950.77	4,099.92	0.101	6,760.89	6,446.09	7,075.69	0.674	12,145.92	12,031.71	12,260.13	0.002
Full-year enrollment	4,126.09	4,031.17	4,221.02		6,637.73	6,129.54	7,145.93		12,549.08	12,319.52	12,778.65	

Notes: Adjusted predicted drug spending were estimated using predictive margins after generalized linear models. *p*-values were obtained from post-estimation Wald tests comparing ACO enrollees and non-ACO beneficiaries. *Abbreviations*: ME, marginal effects; 95% CI, confidence intervals.

Supplemental Table 12. GLM results on Part D drug beneficiary OOP spending among FFS beneficiaries with ADRD and five or more chronic conditions

	FFS only				Dually eligible for 1 to 11 months				Dually-eligible for 12 months			
	MR	95% CI		*p*	MR	95% CI		*p*	MR	95% CI		*p*
ACO (ref: Never/Partial)												
Full-year enrollment	0.97	0.96	0.98	<0.001	1.19	1.07	1.33	0.002	1.34	1.27	1.40	<0.001
Gender (ref: Male)												
Female	0.89	0.88	0.90	<0.001	0.88	0.79	0.98	0.017	0.96	0.92	1.00	0.057
Age (ref: 65-74)												
75-84	0.92	0.90	0.94	<0.001	1.21	1.07	1.38	0.003	0.86	0.81	0.90	<0.001
85 or older	0.80	0.79	0.82	<0.001	1.00	0.87	1.14	0.957	0.63	0.60	0.66	<0.001
Race/Ethnicity												
Black	1.00	0.97	1.03	0.940	0.61	0.52	0.71	<0.001	0.83	0.79	0.88	<0.001
Hispanic	0.87	0.84	0.91	<0.001	0.80	0.64	0.99	0.044	1.04	0.98	1.10	0.198
Other	1.01	0.97	1.05	0.596	0.59	0.47	0.75	<0.001	0.95	0.89	1.01	0.103
Part D coverage												
12-month enrollment	1.74	1.65	1.82	<0.001	9.12	8.04	10.35	<0.001	0.78	0.60	1.01	0.059

Notes: Generalized linear models with a log link and gamma distribution were used to examine Part D drug beneficiary payments, with exponentiated coefficients interpreted as mean ratios. States were controlled but omitted due to limited space. N for FFS only=263,658; N for 1-11 months of dual=13,007; N for 12 months of dual=170,263. *Abbreviations*: ACO, accountable care organization.

	FFS only				Dually eligible for 1 to 11 months				Dually eligible for 12 months			
ACO enrollment	ME	95% CI		*p*	ME	95% CI		*p*	ME	95% CI		*p*
Never/Partial	1,559.07	1,545.61	1,572.53	<0.001	389.01	363.48	414.54	0.003	38.28	37.37	39.19	<0.001
Full-year enrollment	1,512.27	1,496.03	1,528.51		463.40	417.40	509.41		51.13	48.85	53.41	

Notes: Adjusted predicted drug spending were estimated using predictive margins after generalized linear models. *p*-values were obtained from post-estimation Wald tests comparing ACO enrollees and non-ACO beneficiaries. *Abbreviations*: ME, marginal effects; 95% CI, confidence intervals.

Supplemental Table 13. GLM results on Part B drug Medicare payments among FFS beneficiaries with ADRD and five or more chronic conditions

	FFS only				Dually eligible for 1 to 11 months				Dually-eligible for 12 months			
	MR	95% CI		*p*	MR	95% CI		*p*	MR	95% CI		*p*
ACO (ref: Never/Partial)												
Full-year enrollment	1.01	0.96	1.06	0.726	1.19	0.94	1.49	0.146	1.06	0.91	1.23	0.441
Gender (ref: Male)												
Female	0.86	0.82	0.90	<0.001	0.98	0.78	1.22	0.843	0.93	0.82	1.07	0.325
Age (ref: 65-74)												
75-84	0.81	0.76	0.86	<0.001	0.72	0.55	0.94	0.017	0.93	0.79	1.09	0.358
85 or older	0.67	0.62	0.71	<0.001	0.74	0.56	0.96	0.026	0.79	0.67	0.93	0.004
Race/Ethnicity												
Black	0.94	0.85	1.04	0.231	1.25	0.86	1.83	0.245	1.25	1.03	1.51	0.024
Hispanic	0.91	0.80	1.04	0.180	0.61	0.39	0.97	0.038	0.82	0.68	0.99	0.038
Other	0.84	0.74	0.96	0.008	0.87	0.52	1.45	0.583	0.74	0.62	0.90	0.002

Notes: Generalized linear models with a log link and gamma distribution were used to examine Part B drug Medicare payments, with exponentiated coefficients interpreted as mean ratios. States were controlled but omitted due to limited space. N for FFS only=314,551; N for 1-11 months of dual=10,523; N for 12 months of dual=129,098. *Abbreviations*: ACO, accountable care organization.

	FFS only				Dually eligible for 1 to 11 months				Dually eligible for 12 months			
ACO enrollment	ME	95% CI		*p*	ME	95% CI		*p*	ME	95% CI		*p*
Never/Partial	1,552.97	1,507.99	1,597.94	0.726	749.02	658.67	839.37	0.164	937.17	871.38	1,002.96	0.449
Full-year enrollment	1,565.98	1,508.10	1,623.85		887.99	711.12	1,064.86		993.96	856.65	1,131.27	

Notes: Adjusted predicted drug spending were estimated using predictive margins after generalized linear models. *p*-values were obtained from post-estimation Wald tests comparing ACO enrollees and non-ACO beneficiaries. *Abbreviations*: ME, marginal effects; 95% CI, confidence intervals.

Supplemental Table 14. GLM results on Part B drug beneficiary OOP spending among FFS beneficiaries with ADRD and five or more chronic conditions

	FFS only				Dually eligible for 1 to 11 months				Dually-eligible for 12 months			
	MR	95% CI		*p*	MR	95% CI		*p*	MR	95% CI		*p*
ACO (ref: Never/Partial)												
Full-year enrollment	1.00	0.95	1.05	0.945	1.18	0.92	1.51	0.190	1.04	0.89	1.22	0.619
Gender (ref: Male)												
Female	0.86	0.82	0.90	<0.001	0.99	0.78	1.26	0.949	0.93	0.81	1.07	0.303
Age (ref: 65-74)												
75-84	0.80	0.75	0.86	<0.001	0.69	0.52	0.92	0.012	0.93	0.79	1.09	0.375
85 or older	0.66	0.61	0.70	<0.001	0.73	0.55	0.98	0.037	0.79	0.67	0.94	0.007
Race/Ethnicity												
Black	0.95	0.85	1.05	0.300	1.23	0.82	1.86	0.317	1.25	1.02	1.52	0.028
Hispanic	0.92	0.80	1.06	0.243	0.56	0.34	0.91	0.021	0.81	0.67	0.98	0.029
Other	0.83	0.72	0.95	0.006	0.83	0.47	1.44	0.503	0.71	0.58	0.86	0.001

Notes: Generalized linear models with a log link and gamma distribution were used to examine Part B drug beneficiary payments, with exponentiated coefficients interpreted as mean ratios. States were controlled but omitted due to limited space. N for FFS only=314,551; N for 1-11 months of dual=10,523; N for 12 months of dual=129,098. *Abbreviations*: ACO, accountable care organization.

	FFS only				Dually eligible for 1 to 11 months				Dually eligible for 12 months			
ACO enrollment	ME	95% CI		*p*	ME	95% CI		*p*	ME	95% CI		*p*
Never/Partial	365.67	354.36	376.97	0.946	175.68	152.68	198.69	0.209	222.16	205.88	238.44	0.624
Full-year enrollment	365.03	350.64	379.43		207.28	162.73	251.83		231.09	197.81	264.37	

Notes: Adjusted predicted drug spending were estimated using predictive margins after generalized linear models. *p*-values were obtained from post-estimation Wald tests comparing ACO enrollees and non-ACO beneficiaries. *Abbreviations*: ME, marginal effects; 95% CI, confidence intervals.
